# Chemoresistive Gas Sensors Based on Noble-Metal-Decorated Metal Oxide Semiconductors for H_2_ Detection

**DOI:** 10.3390/ma18020451

**Published:** 2025-01-19

**Authors:** Min Zhu, Heng Zhang, Shengming Zhang, Haiyu Yao, Xuerong Shi, Shusheng Xu

**Affiliations:** School of Material Science and Engineering, Shanghai University of Engineering Science, Shanghai 201620, China; m350123105@sues.edu.cn (M.Z.); m355124217@sues.edu.cn (H.Z.); m355123203@sues.edu.cn (S.Z.); m350122116@sues.edu.cn (H.Y.)

**Keywords:** noble metal, metal oxide semiconductors, chemoresistive, H_2_ sensors

## Abstract

Hydrogen has emerged as a prominent candidate for future energy sources, garnering considerable attention. Given its explosive nature, the efficient detection of hydrogen (H_2_) in the environment using H_2_ sensors is paramount. Chemoresistive H_2_ sensors, particularly those based on noble-metal-decorated metal oxide semiconductors (MOSs), have been extensively researched owing to their high responsiveness, low detection limits, and other favorable characteristics. Despite numerous recent studies and reviews reporting advancements in this field, a comprehensive review focusing on the rational design of sensing materials to enhance the overall performance of chemoresistive H_2_ sensors based on noble-metal-decorated MOFs is lacking. This review aims to address this gap by examining the principles, applications, and challenges of chemoresistive H_2_ sensors, with a specific focus on Pd-decorated and Pt-decorated MOSs-based sensing materials. The observations and explanations of strategies employed in the literature, particularly within the last three years, have been analyzed to provide insights into the latest research directions and developments in this domain. This understanding is essential for designing and fabricating highly efficient H_2_ sensors.

## 1. Introduction

Hydrogen(H_2_), serving as both a reducing and carrier gas and a novel energy source, holds immense application value in the chemical, electronics, healthcare, and metal smelting industries. However, H_2_ is susceptible to leakage during its production, storage, transportation, and utilization processes. H_2_ is undetectable by smell due to its lack of color and odor, and high concentrations can cause asphyxiation. Furthermore, when its volumetric concentration in the air falls within the range of 4% to 75%, H_2_ becomes susceptible to explosion upon exposure to an open flame. Consequently, the deployment of H_2_ sensors for detecting H_2_ content in the environment and monitoring its leakage during usage is imperative. Despite advancements in the development of various H_2_ sensors, real-time leak detection and precise localization of leak sources remains challenging due to the fast diffusion of H_2_ [1,2,3,4,5].

An excellent gas sensor should exhibit high responsiveness, fast response/recovery times, robust stability, and exceptional selectivity [6]. Fast response and recovery times for H_2_ detection are essential to achieve real-time monitoring [7]. A low detection limit is also required. For instance, medical diagnosis necessitates a reliable H_2_ gas sensor with a detection limit of approximately 10 ppm to aid healthcare providers in diagnosing specific digestive issues [8].

To develop a highly efficient H_2_ sensor, extensive research efforts have been undertaken and subsequently summarized. Kafil et al. [9] directed their attention towards specific sensor parameters, including sensitivity, selectivity, humidity tolerance, and response time, among others, and proposed corresponding enhancement strategies while analyzing the underlying causes. Sharma et al. [10] concentrated on recent advancements in metal oxide semiconductor (MOS)-based and field effect transistor (FET)-based H_2_ sensors, discussing the pertinent sensing techniques, mechanisms, and factors influencing sensor sensitivity. Kamal Hossain et al. [11] summarized strategies to augment H_2_ sensing performances using noble-metal-decorated nanostructured zinc oxide (ZnO) as sensing materials. Despite the plethora of recent research endeavors and reviews reporting advancements in this field, a comprehensive review specifically focusing on the rational design of sensing materials to enhance the overall performance of chemoresistive H_2_ sensors based on noble-metal-decorated MOSs remains elusive. Herein, we compile observations and explanations of strategies employed in the literature, particularly within the last three years, to provide insights into the latest research directions and developments in this domain. Initially, it introduces the classifications and fundamental operational principles of H_2_ sensors. Following this introduction, the review delves into a comprehensive analysis of the specific operational mechanisms of chemoresistive MOSs-based H_2_ sensors, employing illustrative examples to elucidate these mechanisms. The third section of this review explores the recent progress in the utilization of noble-metal-decorated MOSs for the development of high-performance H_2_ sensors. Ultimately, the review concludes with a concise summation and delineates potential avenues for future research and development.

### Types and Working Principles

**Figure 1:**
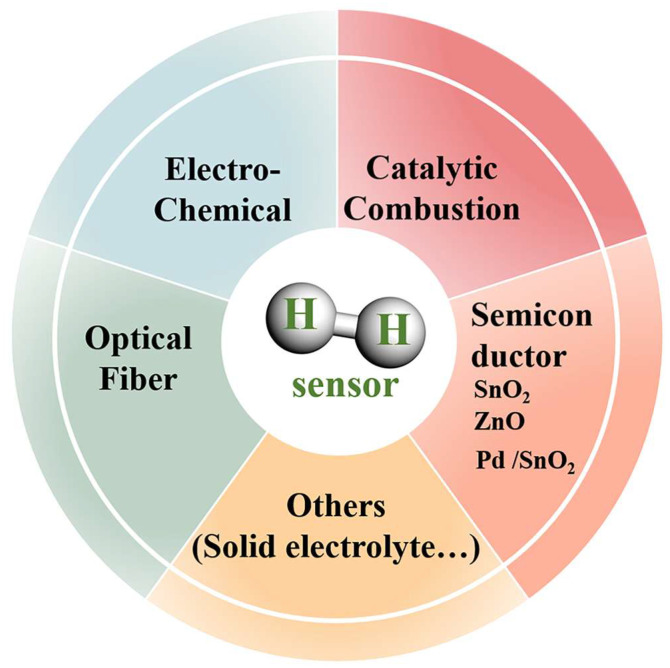
Classification of H_2_ sensors.

H_2_ sensors are primarily categorized into the subsequent classifications (Figure 1).

(1) Electrochemical H_2_ Sensor

An electrochemical H_2_ sensor functions through the utilization of electrochemical reactions. The interaction of H_2_ with the surface of the working electrode elicits variations in the potential of the electrode or adjustments in the circuit current. These modifications can be meticulously measured through the utilization of a reference electrode for calibration, thereby facilitating the precise detection of H_2_ concentration fluctuations. LaConti et al. [12] innovatively deployed a specialized electrochemical sensor in 1971 for the quantitative determination of H_2_ concentrations in gas mixtures containing electrochemically inactive species. After this work, electrochemical H_2_ sensors have undergone significant advancements. In a recent contribution, Wang et al. [13] deliberated on the progression of solid-state electrochemical H_2_ sensors.

(2) Catalytic Combustion H_2_ Sensors

H_2_, characterized as a combustible gas, undergoes a rapid oxidation reaction with oxygen (O_2_), leading to the release of heat. This thermal signal is subsequently converted into an electrical signal via a sensitive transducer element. Depending on the methodology employed for converting thermal signals into electrical signals, these sensors can be further categorized into Pellistor and thermoelectric H_2_ gas sensors. The operational principle of the Pellistor H_2_ sensor involves the generation of heat through a chemical reaction, causing an elevation in temperature of the temperature-sensitive material. This temperature change results in a variation in resistance and, consequently, the creation of a potential difference. In contrast, thermoelectric H_2_ sensors generate electrical signals through the utilization of the thermoelectric effect, also known as the Seebeck effect. In 1985, J.F. McAleer and his colleagues introduced the concept of the thermoelectric H_2_ sensor, thereby laying the theoretical groundwork for subsequent investigative endeavors in the field of thermoelectric H_2_ sensing technologies [14]. More recently, Zhang et al. [15] developed a new catalytic combustion H_2_ sensor using the electrodeposition method.

(3) Optical fiber H_2_ sensors

Optical H_2_ sensors operate by detecting changes in the optical properties of a material upon exposure to H_2_. The utilization of optical fibers as sensing elements in H_2_ sensors was first reported by Butler et al. in the 1980s. The primary advantages of optical fiber H_2_ sensors include their corrosion resistance, suitability for remote sensing, and resistance to electromagnetic interference [16]. Shen et al. [17] has summarized various optical fiber H_2_ sensing technologies since 1984.

(4) Semiconductor-type sensors

This type of H_2_ sensor primarily operates on the basis of the chemical interaction between H_2_ and sensitive materials, which can be readily transduced into an electro-optical signal capable of quantitative assessment. In 1962, Seiyama and colleagues were the first to utilize the unique properties of semiconductor materials for the development of gas sensors [18]. They fabricated gas sensors utilizing zinc oxide (ZnO) semiconductor materials and observed the change of the sensor’s resistance when exposed to reducing gases.

Over the past few years, advancements in material synthesis techniques and the iterative refinement of processing technologies have facilitated the development of high-performance H_2_ sensors. Among them, MOS-based H_2_ sensors boast numerous advantages, including rapid response times, cost-effectiveness, and ease of integration, leading to their extensive application. Various techniques for synthesizing MOSs have been reported, including gas-liquid-solid (GLS) technology [19], electrospinning [20], sol-gel processing [21], hydrothermal synthesis [22], and carbon-thermal transport growth [23]. GLS is commonly used for nanowires, but has complex procedures. Electrospinning is efficient for one-dimensional (1D) nanostructures, but may use toxic solvents. The sol-gel method allows for homogeneous mixing, but may leave residual pores and carbon, requiring heat treatment. Hydrothermal synthesis suits a range of microstructures, but requires precise control. Carbon-thermal transport growth offers specific morphologies and sizes, but may compromise stability and reproducibility. Various MOSs are commonly employed in gas sensing, such as ZnO and tin oxide (SnO_2_), among others.

However, the poor electronic conductivity of MOSs constrains electron transport during gas–solid interactions, and MOSs usually exhibit limited activity for H_2_ detection. To enhance the gas-sensing performance of MOS-based materials, various strategies have been employed, including morphology and size adjustment, exposure of high-energy crystal planes, structural modification, and noble metal decoration. Among these strategies, noble metal decoration was widely employed.

**Figure 2:**
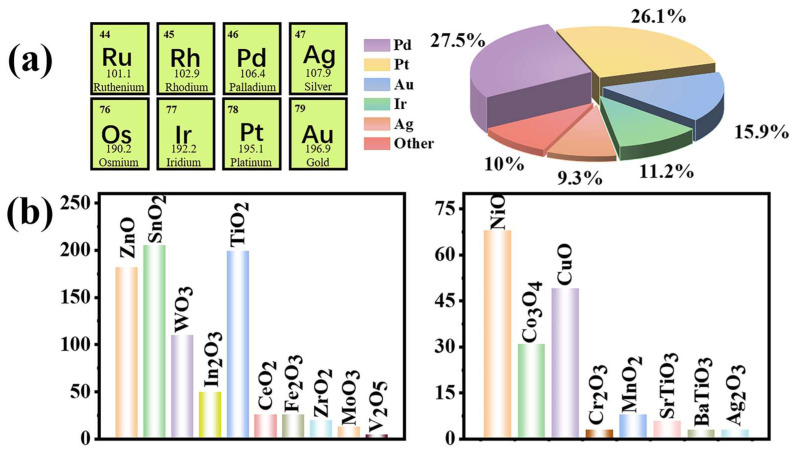
From 2015 to 2024, published articles and patents of noble-metal-decorated MOSs-based H_2_ sensors were categorized: (**a**) based on the type of noble metal utilized, and (**b**) based on the specific MOS employed. Data is from the Web of Science.

In recent years, considerable research has been conducted on noble-metal-decorated MOS H_2_ sensors. As illustrated in Figure 2, over the past decade, the majority of studies on noble metals have concentrated on Palladium (Pd), Platinum (Pt), Gold (Au), Iridium (Ir), and Silver (Ag) for modifying MOSs-based H_2_ sensors. Meanwhile, research on MOSs has primarily focused on n-type semiconductors such as SnO_2_, ZnO, Titanium dioxide (TiO_2_), and tungsten trioxide (WO_3_), as well as p-type MOSs like nickel oxide (NiO), copper oxide (CuO), and Tricobalt tetraoxide (Co_3_O_4_). Numerous studies have demonstrated that the chemical/electronic sensitization of noble metals can substantially enhance the response and selectivity of sensors towards specific gases [24,25,26].

Despite the successful commercialization of MOS-based H_2_ sensors, they operate at relatively high temperatures, ranging from 250 °C to 400 °C, and a significant dependence on humidity. This dependence undermines the reliability of MOS sensors, particularly when operated at room temperature (RT), as noted in [27]. Humidity can interfere with gas–solid interactions by forming water layers, reducing the effective surface area for H_2_ adsorption. High temperatures boost energy consumption and accelerate sensor aging, shortening lifespan. Noble metal decorations can mitigate these issues by inhibiting water adsorption and favoring the adsorption and H_2_ reactions with a low activation energy, enabling low-temperature operation. Moon et al. [28] improved H_2_ detection at RT and reduced humidity impact by modifying SnO_2_ with Pd NPs. Lupan et al. [29] showed enhanced H_2_ detection at RT and a reduced humidity dependence with Au-NP/ZnO NWs, achieving high selectivity and response. To meet the H_2_ sensing performance benchmarks, which include a response time of less than 1 s at a 4% hydrogen concentration and less than 60 s at a 1% H_2_ concentration, along with a recovery time of less than 60 s, it is imperative to develop H_2_ sensors characterized by faster response and recovery performance [30].

## 2. Sensing Mechanism

### 2.1. General Sensing Mechanism of MOSs-Based H_2_ Sensors

The general mechanism of chemical impedance MOSs gas sensing can be elucidated as follows [31]: Firstly, the adsorption of oxygen molecules from the ambient air on the metal oxide surface leads to the trapping of electrons and the conversion of oxygen anions as shown in Equations (1)–(4) [32]. Temperature plays a crucial role in the O_2_ adsorption and dissociation process. As the temperature increases, the O_2_ molecules gain enough energy to undergo chemisorption and capture electrons to form oxygen anions (e.g., O_2_^−^, O^−^, and O^2−^) [33]. At this point, the sensitivity of the sensor increases significantly, but too high a temperature may lead to too fast a reaction between the target gas and the oxygen anions, reducing the selectivity of the sensor [34]. The adsorption and dissociation temperatures of O_2_ vary depending on the sensing material. For example, the reactions of Equations (2)–(4) occur at a temperature below 180 °C, 180 °C~400 °C, and above 400 °C on Pd, respectively. At high temperatures, adsorbed oxygen anions may desorb from the surface, leading to a decrease in the active sites on the sensor surface, thus affecting the sensitivity. Excessive temperatures may also lead to sintering or phase transformation of the MOSs, further degrading the performance of the sensor [27].

(1) $$O_{2}\left(\mathrm{gas}\right)\to O_{2}\left(\mathrm{ads}\right),$$

(2) $$O_{2}\left(\mathrm{ads}\right)+e^{-}\to O_{2}^{-\;}\left(\mathrm{ads}\right),$$

(3) $$O_{2}^{-\;}\left(\mathrm{ads}\right)+e^{-\;}\to \;2O^{-\;}\left(\mathrm{ads}\right),$$

(4) $$O^{-}\left(\mathrm{ads}\right)+e^{-\;}\to O^{2-\;}\left(\mathrm{ads}\right),$$

(5) $$H_{2}\left(\mathrm{gas}\right)\to H_{2}\left(\mathrm{ads}\right),$$

(6) $$H_{2}\left(\mathrm{ads}\right)\to \;2H\left(\mathrm{ads}\right),$$

(7) $$H(\mathrm{ads})+O^{-\;}\left(\mathrm{ads}\right)\to {\mathrm{OH}}^{-\;}\left(\mathrm{ads}\right),$$

(8) $${\mathrm{OH}}^{-\;}\left(\mathrm{ads}\right)+H\left(\mathrm{ads}\right)\to H_{2}O\left(\mathrm{gas}\right)+e^{-},$$

(9) $$4H(\mathrm{ads})+O_{2}^{-\;}\left(\mathrm{ads}\right)\to 2H_{2}O\left(\mathrm{gas}\right)+e^{-\;}$$

In the context of n-type MOSs, such as ZnO and SnO_2_, the presence of an electron depletion layer (EDL) on their surface leads to an increase in surface resistance. Conversely, for p-type MOSs, a hole accumulation layer (HAL) forms on the surface of the sensing material, resulting in a decrease in surface resistance.

When exposed to H_2_, H_2_ molecules adsorb onto the surface of sensing materials, which then reacts with adsorbed oxygen anions present on the MOS surface, as illustrated in Equations (5)–(9). In a H_2_-rich environment, the H_2_ reacts with adsorbed oxygen anions on the surface to produce water, simultaneously releasing electrons back to the material’s surface. For n-type MOSs, this leads to an increase in electron concentration on the outer surface, causing a narrowing of the EDL and resulting in a sensing signal characterized by a reduced resistance value. Conversely, in p-type semiconductors, the emitted electrons decrease the hole concentration, thereby generating a sensing signal marked by an increased resistance value. Furthermore, the specific temperature at which the reactions in Equations (5)–(9) occur is dependent on the sensing material. However, the gas response of conventional MOSs is limited. Therefore, an effective strategy involves the utilization of noble metal nanoparticles for modification.

### 2.2. Sensing Mechanism of Noble-Metal-Decorated MOSs-Based H_2_ Sensors

The modification of MOSs with noble metal nanoparticles, such as Pd, Pt, and Au, represents an efficacious method for enhancing gas response. In comparison to unmodified MOSs, the H_2_ sensing behavior and mechanism of noble-metal-loaded MOSs exhibit a significantly higher degree of complexity. This complexity arises from the intricate interplay between the noble metals and the MOSs, encompassing both surface chemistry and electronic coupling [35].

**Figure 3:**
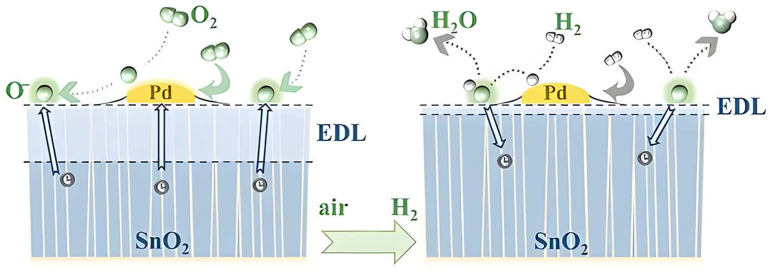
Illustration of the H_2_ gas sensing mechanism of Pd/SnO_2_ [38].

Initially, we focused on the surface chemistry underlying the H_2_ sensing mechanism of noble-metal-decorated MOSs. Extensive research has shown that noble metals serve as catalysts, enhancing the adsorption of oxygen onto material surfaces and facilitating the dissociation of target gas molecules. The Schottky barrier, formed at the junction of noble metal electrodes (Pt, Pd, Au, etc.) and the semiconductor material, is recognized as a critical factor in sensing processes. In the context of H_2_ sensors based on noble-metal-decorated MOSs, beyond the reactions outlined in Equations (1)–(5), there may also be a phenomenon where oxygen and hydrogen atoms diffuse from the noble metal onto the oxide support. This phenomenon is termed “spillover”. Recently, several advanced methodologies have been formulated to gain a deeper understanding of the gas sensing mechanism of noble-metal-decorated MOSs. These methodologies encompass density functional theory calculations and in situ transmission electron microscopy analysis [36,37]. A diverse range of noble-metal-decorated MOSs, including Pd/SnO_2_ and Pt/ZnO, have been employed in H_2_ sensors. Subsequently, we will discuss the sensing mechanism of a typical Pd/SnO_2_ H_2_ sensing system as an illustrative example (Figure 3).

When pure SnO_2_ is exposed to air, O_2_ molecules adsorb onto the SnO_2_ surface. This adsorption process captures free electrons from the conduction band of SnO_2_, forming adsorbed oxygen anions (depending on the operating temperature, as detailed in [39]). Consequently, an EDL forms, leading to an increase in the sensor’s resistance.

When Pd at the nanoscale interfaces with SnO_2_, it extracts electrons from SnO_2_, thereby inducing an EDL at the interfaces [38]. Upon exposure of the Pd/SnO_2_ composite to air, the catalytically active Pd facilitates the dissociation of O_2_ molecules, with the resultant oxygen atoms subsequently diffusing from the Pd to the SnO_2_ support [38] (Figure 3). The diffused oxygen atoms then accept electrons from the bonded SnO_2_, leading to the broadening of the EDL.

When the sensor is subjected to H_2_, as depicted on the right-hand side of Figure 3, H_2_ molecules adsorb onto the Pd surface and disassociate into hydrogen atoms due to the strong affinity of Pd for H_2_ [40]. Subsequently, following spillover, the diffused hydrogen atoms primarily interact with adsorbed oxygen anions [38], forming surface hydroxyl groups [41]. These hydroxyl groups exhibit a reduced electron affinity, causing the release of electrons back to SnO_2_ upon the desorption of produced H_2_O molecules at temperatures exceeding 100 °C. This process results in a significant narrowing of the EDL and a corresponding decrease in its resistance [42]. Note that, following the spillover, the active sites on Pd for O_2_ and H_2_ molecule adsorption and dissociation are freed and become available to capture additional O_2_ and H_2_ molecules, thereby initiating a new reaction cycle.

Moreover, H_2_ can reduce PdO to Pd (Equation (10)), leading to the cessation of electronic interactions or even the formation of a low-work-function Pd hydrid (PdH_x_), which promotes the reverse transfer of electrons [43]. Consequently, the return of a significant number of electrons results in a narrowing of the EDL and a decrease in the sensor’s resistance [44]. The initial reduction reaction of PdO_x_ leads to an increase in the concentration of Pd^0^. This process is considered partial and reversible, as reported in [45]. The resultant reduced Pd can subsequently catalyze the reaction between H_2_ molecules and adsorbed oxygen anions, as outlined in Equations (5)–(9). This catalysis facilitates the release of electrons into the conduction band of SnO_2_, thereby enhancing the conductivity of the composites [46].

(10) $$PdOx+1/2H_{2(g)}\to {Pd}^{0}+x{H_{2}O}_{\left(g\right)}$$

**Figure 4:**
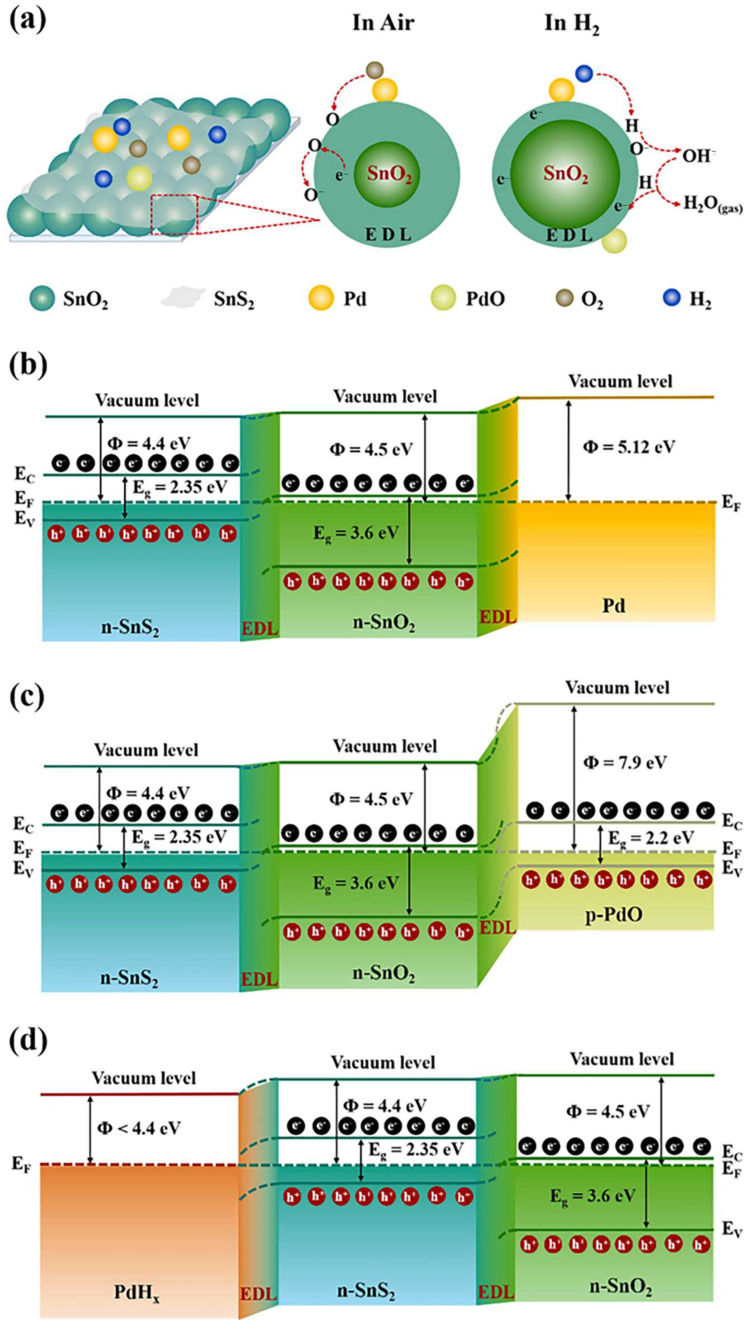
(**a**) Schematic diagram of the Pd/SnS_2_/SnO_2_ H_2_ sensing mechanism. The band structure of (**b**) Pd/SnS_2_/SnO_2_, (**c**) PdO/SnS_2_/SnO_2_ and (**d**) PdH_x_/SnS_2_/SnO_2_ [47]. Reprinted with permission from Elsevier, copyright 2022.

The aforementioned mechanism elucidates the superior sensing performance of noble-metal-decorated MOSs at both room and high temperatures. Notably, Pd has been documented as an exceptionally efficient catalyst for H_2_ dissociation, even at low temperatures [32]. Meng et al. [47] proposed that, in addition to the adsorption of H_2_ and O_2_ on Pd surfaces, electron sensitization of Pd can also facilitate the redistribution of interfacial electrons (Figure 4a). Considering the example of 1.0 at% Pd/SnS_2_/SnO_2_, the metal-semiconductor interface exhibits distinct phenomena due to the difference in work functions. Specifically, the work function of Pd is higher than that of the SnS_2_/SnO_2_ semiconductor (Figure 4b). Consequently, the energy band in the semiconductor shifts downward, resulting in the formation of Schottky barriers at the metal–semiconductor interface. Within the 1.0 at% Pd/SnS_2_/SnO_2_ composition, Pd exists not only in its metallic form, but also as PdO. Notably, the work function of PdO (Ф = 7.9 eV) is higher than that of SnS_2_/SnO_2_, prompting electrons to transfer from SnS_2_/SnO_2_ to PdO, leading to the formation of a p–n heterojunction (Figure 4c). The concurrent generation of Schottky barriers and p–n heterojunctions broadens the EDL in SnS_2_/SnO_2_, thereby increasing the baseline resistance (*R*_a_ ≈ 225 MΩ). Upon exposure of Pd/SnS_2_/SnO_2_ to H_2_, a portion of Pd converts to PdH_x_, characterized by a lower work function (φ < 4.4 eV, Figure 4d). This shift causes electrons to flow from PdH_x_ back to SnS_2_/SnO_2_, increasing the electron concentration in SnS_2_/SnO_2_ and subsequently decreasing the resistance (*R*_g_) of the Pd/SnS_2_/SnO_2_ material. These synergistic effects result in a significant variation in resistance and contribute to the excellent sensing characteristics of the material.

The small amount of Pd could exhibit three distinct functionalities: catalyzing the dissociation of O_2_ molecules, catalyzing the dissociation of H_2_ molecules, and exerting a direct influence on the thickness of the EDL. Among these functionalities, the primary role of Pd is likely to serve as a catalyst for H_2_ dissociation, attributed to its efficient capability to facilitate this process even at a low temperature—a pivotal advantage in the context of H_2_ sensing applications. Nevertheless, the significance of the other two roles should not be overlooked. When Pd is deposited onto MOSs, it can further influence the sensor’s response characteristics via complex interactions between Pd and the MOSs. These interactions encompass various facets, including interface chemistry and electronic coupling mechanisms, which collectively govern the overall performance of the sensor.

In summary, due to the interaction between noble metals and MOSs, the H_2_ sensing mechanism of noble-metal-loaded MOSs is complex, and many aspects such as surface chemistry and electronic coupling need to be considered.

## 3. Noble-Metal-Decorated MOSs-Based Gas Sensors

### 3.1. Pd-Decorated MOSs-Based Gas Sensors

Pd-decorated MOSs exhibit heightened sensitivity, remarkable selectivity, fast response/recovery times, and low detection limits for H_2_ sensing, attributable to the distinct solubility of H_2_ in Pd and its capacity to form PdHx. Notably, only H_2_ is capable of inducing significant lattice expansion in Pd, due possibly to the small radius of the hydrogen atom, whereas other gases such as carbon monoxide (CO) exhibit no such effect. This specificity contributes to the excellent selectivity of the Pd-based sensor. The processes of hydrogen adsorption and desorption on Pd occur rapidly, facilitating fast response and recovery. Furthermore, the rapid diffusion and high solubility of hydrogen atoms within the Pd lattice result in substantial resistance changes even at trace concentrations of H_2_, enabling low detection limits.

Given these advantages, Pd catalysts are extensively utilized for H_2_ detection. Various studies have shown that the reduction of Pd precursors can be accomplished through solution-based methods involving UV light irradiation [48], chemical reducing agents [49], and thermal treatments [50].

#### 3.1.1. Pd-Decorated SnO_2_

Among the diverse array of semiconductors, n-type SnO_2_, characterized by its wide band gap of 3.5~4.0 eV [51,52], which can be measured via photocurrent spectroscopy [53], stands out as a promising candidate for sensing applications due to its low cost, simple manufacturing technique, and good long-term stability. However, its application is hindered by limitations such as low sensitivity, high operational temperature, inadequate selectivity, and sluggish response kinetics. To overcome these disadvantages, the modification of SnO_2_ through the incorporation of other materials, particularly noble metals such as Pd, has proven to be an effective strategy for improving its sensing performance.

Manipulating the dimensionality [54,55,56,57] and dispersion of Pd on MOSs supports can fine-tune their H_2_ sensing performances. So far, a number of Pd-based H_2_ sensors, including nanowires (NWs) [5,58,59], nanosheets (NSs) [60], nanofibers (NFs) [59,61], nanoflowers [62], nanorods (NRs) [63,64], nanotubes (NTs) [65,66], and films have been suggested [47].

**Figure 5:**
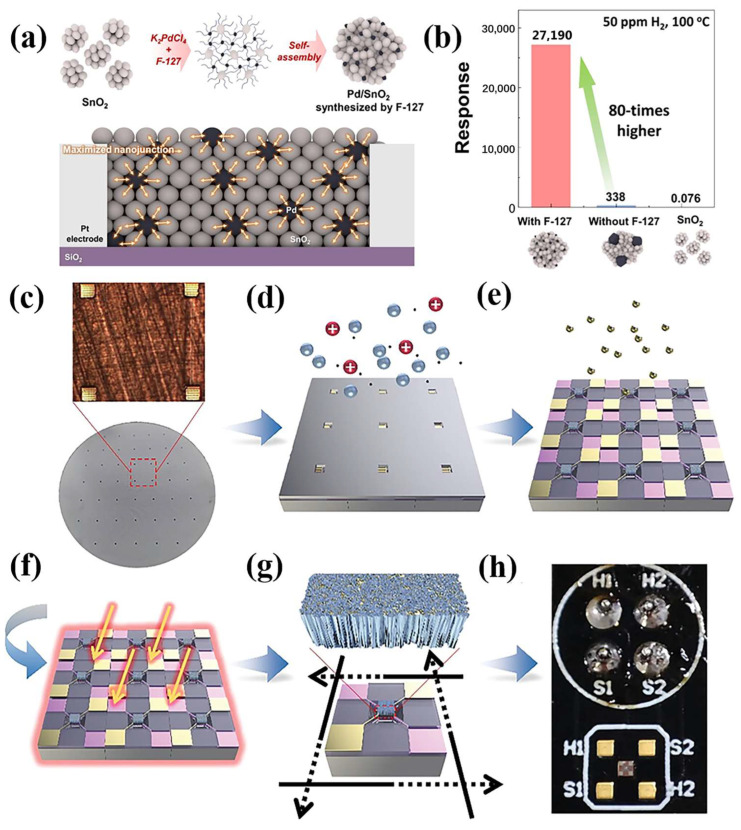
(**a**) Schematic illustration of the synthesis mechanism of F-Pd/SnO_2_ nanoparticles with Pluronic F-127 assistance and corresponding response comparison plot of SnO_2_, Pd/SnO_2_ [69]. Reprinted with the permission from Elsevier, copyright 2024. (**b**) F-Pd/SnO_2_ to 50 ppm of H_2_ at 100 °C [69]. (**c**) Schematic of the fabrication of Pd/SnO_2_ sensing film patterns and MEMS H_2_ sensing chips: The micro hotplate arrays are aligned with the mask [38], (**d**) SnO_2_ film patterns are deposited in the central sensing area by a mask-assistant magnetron sputtering method [38], (**e**) Pd NPs catalysts are further decorated on SnO_2_ by ALD [38], (**f**) the film patterns perform annealing treatment in air-H_2_-air, and finally the MEMS H_2_ sensing chips are obtained after (**g**) dicing and (**h**) wire bonding [38].

The nanoparticles (NP) enhance sensor sensitivity by providing a larger surface area for gas adsorption. Nam et al. [67] (Table 1) fabricated Pd/SnO_2_ nanoparticles (NPs) for an exceptionally sensitive and selective H_2_ gas sensor by leveraging Pluronic F-127 (Figure 5). Pluronic F-127, a block copolymer structured as (polyethylene oxide)99-(polypropylene oxide)69-(polyethylene oxide)99 [68], features three hydrophilic chains and a central hydrophobic chain. It functions dualistically as a reducing agent and surfactant, enhancing the dispersion of Pd NPs and modulating their size. In comparison to pristine SnO_2_ and Pd/SnO_2_ NPs synthesized without the aid of F-127, the Pd/SnO_2_ NPs synthesized with F-127 assistance, denoted as F-Pd/SnO_2_, exhibited a superior H_2_ response of 27, 190 and a fast response time of 3 s when exposed to 50 ppm of H_2_ at 100 °C (Figure 5). This enhancement is attributed to the increased number of nanojunctions.

In the above work and some other literature [47], H_2_ sensors utilizing SnO_2_ nanostructures exhibit linear response characteristics within a concentration range of up to 1% H_2_. Only a few works reported H_2_ sensing properties at concentrations exceeding this threshold. For H_2_ concentrations ranging from 1% to 2%, detection utilized the volumetric expansion characteristic of the β-phase PdH_x_. This expansion arises from the phase transition from α-Pd to β-PdH_x_, causing the reconnection of previously disrupted junctions within the Pd, thereby resulting in a fast decrement of the resistance in Pd-based sensors [69].

**Table 1:** The comparison of sensing performances of noble-metal-decorated MOSs.

Materials	Optimal Temperature (°C)	τ_res_/τ_rec_ (s)	Detection Limit (ppm)	Concentration (ppm)	Response	Ref.
F-Pd/SnO_2_	100	3/NA ^a^	50	50	27,190 ^c^	[67]
30Pd/SnO_2_	150	NA/30 ^a^	0.5	20	1.51 ^d^	[38]
Pd/SnS_2_/SnO_2_	300	1/9 ^a^	10	500	90 ^d^	[47]
Pd/SnO_2_	210	3.4/5.6 ^a^	1.5	5000	712.65 ^e^	[35]
SnO_2_-Pd@rGO	390	8/3 ^a^	0.1	200	243.5 ^f^	[32]
NiO-shelled Pd-decorated ZnO NW	200	NA	NA	100	13.36 ^d^	[62]
Pd@ZnO-2	350	84/468 ^a^	NA	100	22 ^d^	[70]
Pd/Fe_2_O_3_-NiO NFs	250	11/105 ^a^	1	1000	199.24 ^d^	[71]
*p*-PdO-*n*-WO_3_-heterostructure film	160	4/NA	0.5	100	45.1 ^d^	[72]
Pt SnO_2_–Co_3_O_4_	300	12/NA	NA	100	57.9 ^d^	[73]
Pd-doped rGO/ZnO-SnO_2_	380	4/8 ^a^	9.4	100	9.4 ^d^	[74]
5.0 wt% Pd NPs/CeO_2_-C	25	3/NA ^a^	NA	100	1322 ^d^	[75]
Pt-TiO_2_-MoS_2_	100	NA	50	500	47.09 ^d^	[76]
Pt-SnO_2_	25	13/NA ^a^	NA	1000	5000 ^e^	[77]
Pt-SnO_2_	825	NA	NA	1000	450 ^e^	[78]
ZnO–Pt	300	133/112 ^a^	100	1000	132.5 ^g^	[79]
WO_3_/Pt-ZnO	R.T.	19/81^a^	1	100	61.5 ^d^	[80]
ZNT/G	R.T.	30/38 ^a^	10	100	28.08 ^d^	[81]
MoS_2_-HIZNTs	R.T.	14/19 ^a^	10	500	51.1 ^h^	[82]
Pt–Fe_2_O_3_–V_o_	240	2/45 ^b^	0.086	50	NA	[83]
PtRu/CeO_2_	500	97/123 ^a^	100	100	NA	[84]
Ag@SnO_2_@g-C_3_N_4_	300	¾ ^a^	0.03	50	5.4 ^d^	[85]
Ir_red_/ZnO-450	450	7/9.7 ^a^	10	100	5.5 ^d^	[86]

^a^: The response time (τ_res_) and the recovery time (τ_rec_) were determined as the time taken for the resistance (current) to reach 90% of the saturated response upon exposure to H_2_ and to decrease by 90% back to baseline after removal, respectively. ^b^: $\tau =\upsilon_{0}^{-1}\exp\left(\frac{{-E}_{ads}}{TK_{B}}\right)$. υ_0_, K_B_, and T are the attempt frequency, Boltzmann constant, and temperature. ^c^: R = (R_a_ − R_g_)/R_g_. R is the sensor response. R_a_ and R_g_ are the sensor resistance in air and target gas, respectively. ^d^: R = R_a_/R_g_. ^e^: △I/I_0_ = [(I − I_0_)/I_0_] × 100%. I and I_0_ represent the current values under target gas and baseline (no target gas) conditions, respectively. ^f^: R = I_g_/I_0_. ^g^: R = (G_g_ − G_a_)/G_a_ × 100%. G_g_ and G_a_ are the conductance of H_2_ gas and air stimulation at different ppm of the sensor, respectively. ^h^: R = R_a_/R_g_ × 100%. NA: Not available.

A comprehensive elucidation of the H_2_-sensing mechanism at concentrations above 1% is required. Liu et al. (Table 1) have proposed the existence of two H_2_ concentration-dependent sensing mechanisms for their developed Pd/SnO_2_ NPs film-based H_2_ sensor, designed for H_2_ detection across a wide concentration range (1.5 ppm to 10%) [35]. Specifically, below a 1% H_2_ concentration, the sensor response exhibits a linear correlation with the square root of the H_2_ concentration, primarily attributed to the electronic coupling effect occurring at the interface between PdH_x_ and SnO_2_. This mechanism facilitates a high sensitivity of 0.23 ppm⁻¹. As the H_2_ concentration increases beyond this point, a linear dependence between the response and H_2_ concentration is observed, with a sensitivity of 0.018 ppm⁻¹. This latter behavior is attributed to the redox reaction between H atoms and the adsorbed oxygen anions on the SnO_2_ surface [35].

SnO_2_-based H_2_ sensors show a high compatibility with integrated circuits (IC). The development of intelligent and integrated H_2_ sensors has emerged as a focal area of contemporary research. As gas sensing chips transition into mass production, a multitude of challenges have come to light. Achieving a uniform film at the wafer level is a pivotal prerequisite for ensuring high consistency among sensing chips. However, conventional techniques such as drop-coating and screen-printing fall short at meeting this criterion due to their inherent lack of precision and uncontrollability. Furthermore, these methods often lead to unwanted contact between sensing materials and electrode pads, thereby inducing signal crosstalk between the heating and testing electrodes, and potentially compromising the subsequent packaging process. Consequently, the development of effective gas sensing film patterning methods and noble metal catalytic modification techniques at the wafer level is of paramount importance for the mass manufacturing of sensing chips. Recently, Zhang et al. [38] (Table 1) have devised a straightforward methodology that integrates atomic layer deposition (ALD), magnetron sputtering, and subsequent annealing in an air-H_2_-air atmosphere to fabricate high-performance Pd/SnO_2_ film patterns tailored for H_2_ sensing (Figure 3). This approach allows for precise regulation of the grain size and crystallinity of the Pd/SnO_2_ films through meticulous control of the deposition and annealing processes, ultimately enhancing their H_2_ sensing capabilities. The resultant MEMS H_2_ sensing chips exhibit remarkable consistency and a broad detection range spanning from 0.5 to 500 ppm. Notably, even at an H_2_ concentration as low as 0.5 ppm, a discernible change in resistance and response value (with a signal-to-noise ratio exceeding 3) is observed [38]. This sensing chip boasts a lower detection limit and an expanded detection range encompassing three orders of magnitude compared to certain previously reported H_2_ sensors [38]. Two problems may be encountered regarding thin films such as film uniformity and signal crosstalk, which can be further explored with more advanced deposition techniques, as well as with new patterning methods. A combination of deposition techniques and patterning methods can be used to significantly improve the performance of H_2_ sensors. For example, ALD deposits a uniform film and combines it with an isolation trench design to reduce signal crosstalk or deposit a uniform catalytic layer by sputtering. Adding a shielding layer also blocks electric field interference [87].

**Figure 6:**
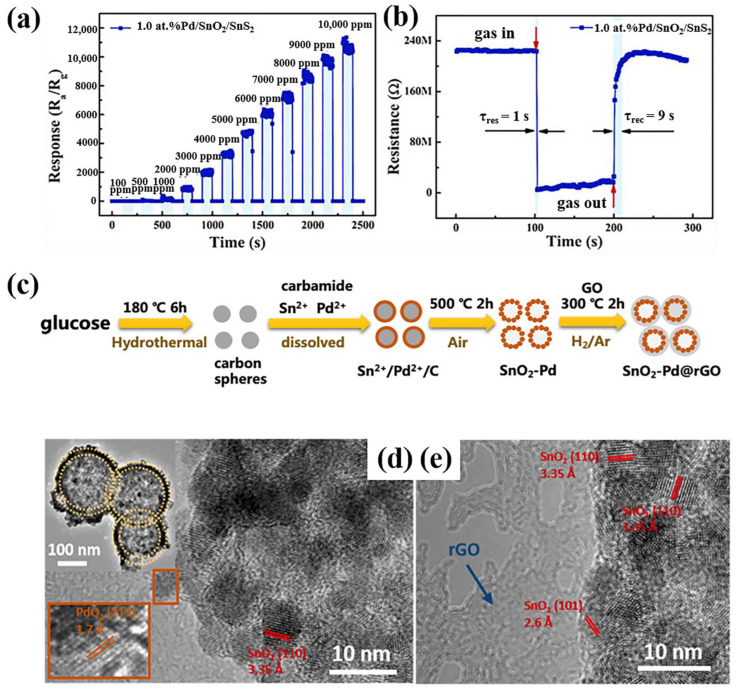
(**a**) Response curves and (**b**) response/recovery time of Pd/SnS_2_/SnO_2_ towards 500 ppm H_2_ [47]. Reprinted with permission from Elsevier, copyright 2022. (**c**) The schematic for the rGO-wrapped SnO_2_–Pd hollow porous spheres. SnO_2_–Pd@rGO: (**d**) TEM image (inset: high-resolution HRTEM image), (**e**) HRTEM image [32]. Reprinted with permission from Elsevier, copyright 2022.

In addition to the decoration with noble metals, the further modification of SnO_2_ with alternative semiconductors offers a viable approach to enhancing its H_2_ sensing capabilities, primarily due to the substantial resistance modulation at heterojunction barriers. Various semiconductors have been utilized to establish heterojunctions with SnO_2_ and are co-supported with noble metals [88]. Notably, 2D semiconductors have emerged as prominent candidates owing to their layered structure, high surface-to-volume ratio, unique semiconducting attributes, and substantial electronegativity [89]. Tin disulfide (SnS_2_), for instance, exhibits considerable potential in heterostructure-based sensing applications. Meng et al. (Table 1) synthesized SnO_2_@SnS_2_ hollow nanostructures through a combined hydrothermal and impregnation approach [47]. The optimized 1.0 atomic percent (at%) Pd/SnS_2_/SnO_2_ nanocomposites exhibited a peak response of 95 towards 500 parts per million (ppm) H_2_ at 300 °C (Figure 6a), which was 10.6 times higher than that of pure SnO_2_ nanoparticles and 5.3 times higher than that of pure SnS_2_/SnO_2_-2 nanocomposites [47]. Furthermore, the 1.0 at% Pd/SnS_2_/SnO_2_ composites demonstrated rapid response and recovery times of 1 and 9 s (Figure 6b), respectively, along with exceptional selectivity and stability [47]. The enhanced H_2_ sensing properties of the Pd/SnS_2_/SnO_2_ nanocomposites may be attributed to several factors: (1) the spillover effect of Pd, (2) the formation of a Schottky barrier at the interface between Pd and SnS_2_/SnO_2_, and (3) the establishment of a p-n heterojunction at the junction between PdO and SnS_2_/SnO_2_.

Apart from SnS_2_, graphene and reduced graphene oxide (rGO) have been extensively employed to construct heterojunctions with MOSs to achieve impressive gas sensing performances, attributed to their large specific surface areas and exceptional electron mobility [32]. Notably, the hydrophobicity of rGO has been confirmed to suppress the effects of high humidity on graphene-based gas sensing [90]. Qiu et al. (Table 1) fabricated an rGO-encapsulated SnO_2_–Pd porous hollow sphere composite (SnO_2_–Pd@rGO) for a high-performance H_2_ sensor [32]. The porous hollow architecture of this composite was derived from a carbon sphere template (Figure 6c). The encapsulation with rGO was achieved through the self-assembly of GO onto SnO_2_-based spheres, followed by thermal reduction in a H_2_ atmosphere (Figure 6c). This sensor demonstrated outstanding selective H_2_ sensing characteristics at 390 °C, exhibiting a linear response across a broad concentration range (0.1–1000 ppm) with a fast recovery time of 3 s. It also showed a high response of approximately 8 to 0.1 ppm H_2_ within one minute and maintained acceptable stability under high humidity conditions (e.g., 80%). The calculated detection limit of 16.5 ppb facilitated the potential for trace H_2_ monitoring. Furthermore, the sensor displayed a detectable response to H_2_ at a minimum concentration of 50 ppm at 130 °C. These remarkable performances were primarily attributed to the unique hollow porous structure with abundant heterojunctions (Figure 6d,e), the catalytic activity of doped-PdO_x_, the relatively hydrophobic surface provided by rGO, and the deoxygenation process following H_2_ reduction.

The chemiresistive gas sensing mechanism of the MOSs composite is attributed to both electronic sensitization (i.e., energy band modulation and heterojunction formation) and chemical sensitization (i.e., doping spillover and oxygen adsorption) [91]. In the context of electronic sensitization, heterojunctions are established between p-type rGO and n-type SnO_2_. The disparity in their work functions (5.1 eV for rGO and 4.5 eV for SnO_2_) [92] leads to the formation of an electron depletion region on the SnO_2_ side at the SnO_2_/rGO interface.

Notably, the experimental evidence revealed that the SnO_2_–Pd@rGO composite predominantly comprises PdO and PdO_2_ phases, accounting for 96.1% of the total Pd content. The doped PdO_x_ species are recognized as potent electron acceptors exhibiting p-type behavior, which can effectively lower the Fermi level of SnO_2_. Consequently, an additional electron depletion region is induced on the SnO_2_ side at the SnO_2_/Pd interface. Furthermore, PdO_2_, being metastable and more reactive towards the target gas compared to PdO [93], is likely advantageous for enhancing the H_2_ sensing performance of the SnO_2_–Pd@rGO composite, given the substantial presence of PdO_2_ in the material.

#### 3.1.2. Pd-Decorated ZnO

As discussed above, SnO_2_ exhibits high conductivity and remarkable sensitivity to low gas concentrations, particularly enabling a swift response to variations in H_2_ concentration and generating pronounced electrical signal changes in H_2_ sensors. However, ZnO offers several advantages over SnO_2_ in the context of H_2_ sensors utilizing MOSs. Specifically, ZnO boasts superior biological adaptability, safety, unique piezoelectric properties, and potential fabrication process and cost benefits. Consequently, in certain specific applications, ZnO may be a more suitable sensing material for H_2_ sensors.

Nonetheless, the sensing application of pure ZnO is constrained by its low response, instability, and particularly poor H_2_ selectivity. To address these limitations, functionalizing ZnO with noble metal catalysts, such as Pd and Pt, presents a promising approach to enhance the properties of the material [70].

The presence of relatively high humidity leads to a decreased gas response in ZnO sensors, owing to competition for adsorption sites on the sensor surface between H_2_O molecules and H_2_ molecules [94]. Nevertheless, the incorporation of noble metal NPs has been found to enhance the stability of gas sensors under humid conditions [95].

**Figure 7:**
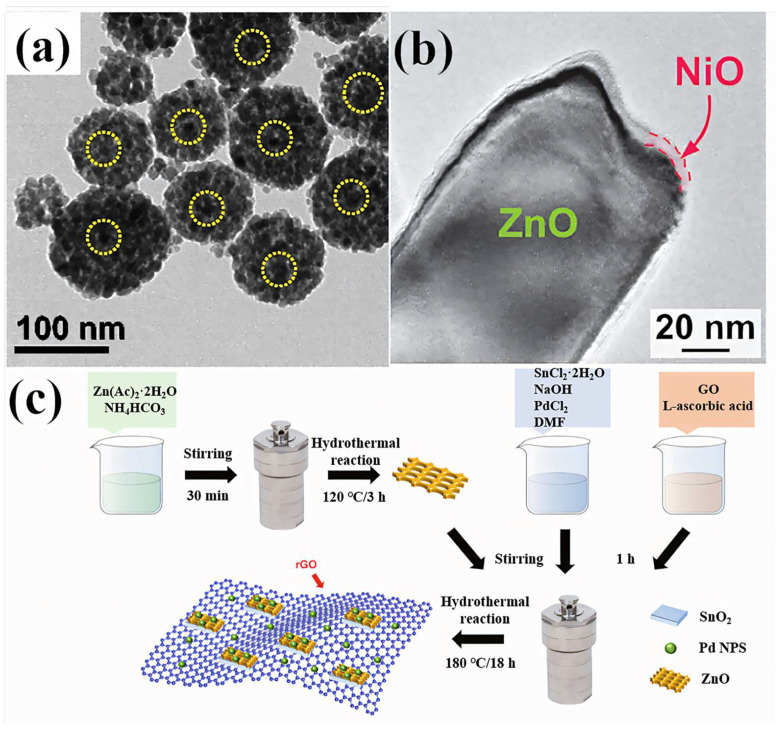
(**a**) TEM observation for Pd@ZnO-2 [70]. Reprinted with the permission from Elsevier, copyright 2020. (**b**) Transmission electron microscope (TEM) image of a NiO-shelled Pd-decorated ZnO NW [60]. Open access. (**c**) The fabrication process of the Pd-doped rGO/ZnO-SnO_2_ nanocomposites [74]. Open access.

To enhance the sensing performance by generating abundant active sites, effective approaches involve forming MOSs heterojunctions and modifying the morphology to achieve a larger specific surface area. Controlling the morphology of Pd–ZnO structures can regulate their H_2_ sensing capabilities. For instance, Nguyen et al. (Table 1) observed the microscopic morphology of Pd@ZnO-2 using TEM (Figure 7a) [70]. The Pd-decorated ZnO showed improved H_2_ sensing performances compared with the ZnO NPs.

However, the operation of gas sensors at high temperatures can lead to the oxidation of ultrafine noble metal particles in the air, thereby reducing sensing performance and causing agglomeration of noble metals on the sensor surface [96]. Therefore, it is crucial to devise a method to shield noble metals from direct air exposure to enhance sensing performance. One effective preservation strategy involves depositing a thin layer of metal oxide on a surface adorned with nano-noble metal particles. Among the candidates for protective layer materials, p-type NiO has garnered significant attention due to its robust structural stability, exceptional oxygen adsorption capacity, and potent catalytic activity [97]. Badie et al. (Table 1) [60] employed NiO as a deposition layer to coat Zn NWs decorated with Pd (Figure 7b). In the absence of Pd decoration on ZnO, NiO comes into direct contact with ZnO. The intimate contact between n-type ZnO and p-type NiO generates numerous heterojunctions with potential barriers that impede charge flow in air. Upon exposure to the target gas, the barrier height alters, leading to a change in sensor resistance. Since NiO is deposited as a continuous layer, a core-shell structure forms, with ZnO and NiO in direct contact, maximizing the contact area between the components and resulting in substantial resistance variations. Alternatively, when Pd nanoparticles are positioned between ZnO and NiO layers, Zn-Pd-Ni oxide heterojunctions are established [60]. Compared to single-component materials, Zn-Ni oxide heterojunctions exhibit superior performance in gas sensing applications [98].

P-type MOSs, exemplified by NiO as previously mentioned, offer distinct advantages over n-type MOSs in the selectivity and monitoring of reducing gases. This superiority is attributed to the extensive adsorption of oxygen on their surfaces [71]. Research has demonstrated that sensors based on noble-metal-decorated NiO exhibit promising sensing capabilities for H_2_ detection [99]. Cai et al. (Table 1) [71] fabricated porous NiO NFs embedded with Pd and Fe_2_O_3_ NPs through a straightforward electrospinning process. These sensors achieved a maximum response value of 199.24 for 1000 ppm H_2_ gas at an optimal operating temperature of 250 °C, accompanied by a response/recovery time of 11/105 s. Additionally, they exhibited robust selectivity and long-term stability towards H_2_. The exceptional gas sensing performance of these sensors is primarily attributed to the catalytic effect of Pd and the unique fluffy porous 1D microstructure, which features tightly linked p-n heterojunctions between NiO and Fe_2_O_3_. This structure provides a large specific surface area and numerous active sites, thereby facilitating the reaction between H_2_ molecules and surface oxygen anions.

Similarly, Zhu et al. (Table 1) [72] observed that Pd in Pd-decorated WO_3_ composites exists in the form of PdO, forming a p-n heterojunction. The H_2_ gas sensor assembled using the p-PdO-n-WO_3_ heterostructure and a uniformly dispersed thin film exhibited excellent sensing performance, high sensitivity, a low detection limit, and good stability. At an optimal operating temperature of 160 °C, the sensor demonstrated response values (R_a_/R_g_) of 1.2 and 45.1 for H_2_ concentrations of 500 ppb and 100 ppm, respectively. Furthermore, the response times were 38 s and 4 s for these concentrations.

In addition to the construction of p-n heterojunctions, the H_2_ sensing performance of materials can also be improved by constructing n-n heterojunctions and/or adding other components. For example, Zhang et al. (Table 1) [74] prepared quaternary nanocomposites (Figure 7c) by hydrothermal method using Pd-doped rGO/ZnO-SnO_2_ for use as sensing materials in H_2_ sensors. Compared with ZnO-SnO_2_ composites, the materials doped with 3 wt% rGO (NC3) exhibited a better H_2_ response. The maximum H_2_ response of the NC3 material at 380 °C is 9.4, which is two times that of NC0, i.e., ZnO-SnO_2_.

**Figure 8:**
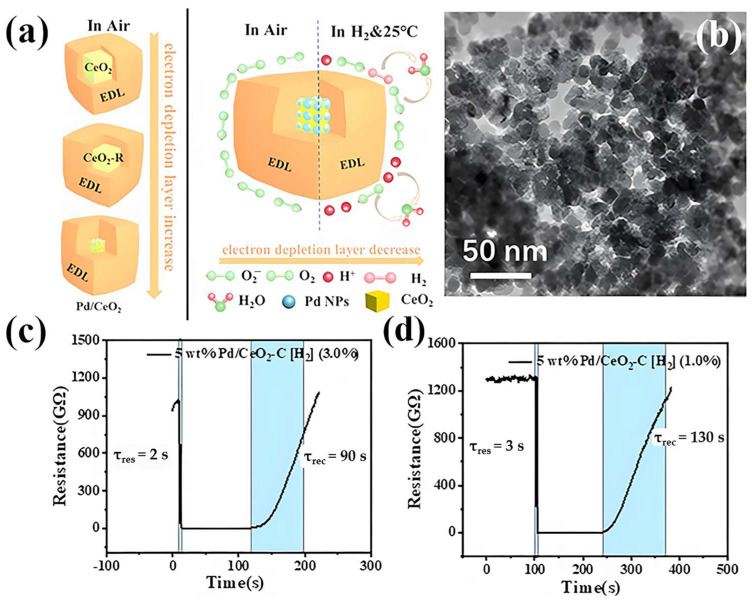
(**a**) Mechanistic diagram of the 5 wt% Pd NPs/CeO_2_-C sensor [75]. (**b**) TEM images of 5 wt% Pd NPs/CeO_2_-C5 wt% Pd NPs /CeO_2_-C [75]. (**c**) Variation of response times of different proportions of 5 wt% Pd NPs CeO_2_ at 25 °C in 10,000 ppm H_2_ in 1% and (**d**) 3% H_2_ concentration [75]. Reprinted with permission from Elsevier, copyright 2023.

Oxygen vacancies, a prevalent and crucial type of crystal defect, play a significant role in the sensing performance of these semiconductors [100]. Various strategies have been proposed to enhance the oxygen vacancy content. Traditional methods, such as high-temperature gas reduction and calcination, are commonly employed to create oxygen defects [56,101,102,103,104,105,106]. However, these methods often require complex or hazardous conditions, including high temperatures and H_2_-rich atmospheres. Consequently, the use of an appropriate reducing agent to regulate the concentration of oxygen vacancies at RT has become increasingly important. Ascorbic acid, for instance, serves as an effective reducing agent, promoting the formation of both surface and intrinsic hydroxyl groups. The hydroxyl-oxygen vacancy model introduces a novel mechanism for the generation of oxygen vacancies, wherein hydroxyl groups and oxygen vacancies coexist, with the latter providing accommodation space for adjacent hydroxyl groups. Song et al. (Table 1) [75] developed a “hydroxyl-oxygen vacancy model” utilizing the redox-capable Ce^4+^ ↔ Ce^3+^ system (Figure 8). Following ascorbic acid reduction, Pd NPs-modified cerium dioxide (CeO_2_) (in cubic, Figure 8b, rod-shaped, and spherical morphologies) exhibits a high abundance of hydroxyl groups [75]. This approach not only facilitates the formation of oxygen vacancies within the CeO_2_ lattice, but also establishes a linear correlation between the surface Ce^3+^ content, the content of oxygen vacancies, and highly reactive oxygen species [75]. The optimal 5.0 wt% Pd NPs/CeO_2_-C, characterized by the highest concentration of oxygen vacancies and Ce^3+^ content, owned the largest EDLs in air (Figure 8a). It demonstrated rapid sensing kinetics (3 s for 1% H_2_ and 2 s for 3% H_2_, Figure 8c,d) and remarkable sensitivity to H_2_ (*R*_a_/*R*_g_ of 1322 for 1% H_2_), with a detection limit as low as 50 ppm [75].

### 3.2. Pt-Decorated MOSs

Pt is also widely used for the H_2_ detection. Compared with Pd, Pt has a lower affinity for H_2_ molecules, is less prone to H_2_ embrittlement, and has better long-term stability. And under certain extreme conditions (e.g., high temperature, strong acid and alkali environment), Pt may exhibit higher stability. Due to the high catalytic activity of Pt, Pt can quickly promote the reaction of H_2_ with the sensor surface. Therefore, when Pt is used as a catalyst, the response speed of the H_2_ sensor is usually faster, and when the H_2_ concentration decreases, the sensor doped with Pt is usually able to return to its initial state more quickly. Accordingly, Pt-decorated MOSs-based H_2_ sensors show great potential in applications that require high sensitivity, high selectivity, and a fast response.

#### 3.2.1. Pt-Decorated SnO_2_

In practical applications within the realm of gas sensors, SnO_2_-based gas sensors continue to be a leading choice. Various strategies have been employed to enhance the gas sensitivity of Pt-decorated SnO_2_-baed H_2_ sensors, including the construction of n-n, p-n, and p-p heterojunction composites [107,108,109], the design of hierarchical structures, and the addition of catalytic layers. Luo et al. (Table 1) [76] successfully synthesized a ternary Pt–TiO_2_/MoS_2_ composite through a two-step hydrothermal method, combining TiO_2_ nanoparticles with flower-like MoS_2_ structures and depositing Pt. The optimal composites exhibited remarkable sensitivity and selectivity towards H_2_ at 100 °C. Similarly, Yin et al. (Table 1) [73] prepared SnO_2_–Co_3_O_4_ p-n heterojunction-based Pt sensing materials via a hydrothermal method, which demonstrated excellent gas sensitivity and selectivity for H_2_ at an optimal operating temperature of 300 °C with an optimal amount of 5% Co.

The long-term stability of MOSs-based sensing materials at RT is crucial for H_2_ sensors, as it represents a significant hurdle to their commercialization. Huang et al. (Table 1) [77] addressed this challenge by preparing 1 wt% Pt-doped Pt–SnO_2_ nanocomposites that exhibit impressive room-temperature H_2_ sensing capabilities. However, these capabilities diminished rapidly over time. Specifically, after seven days of aging, the response to 1% H_2_ at RT decreased by a factor of 50. Notably, gentle heat treatment (e.g., 10 min at 140 °C) fully restored the room-temperature H_2_ sensing performances of the aged sample. In contrast, the robust response of the Pt–SnO_2_ nanocomposite with 5 wt% Pt to 1% H_2_ at RT, synthesized by Zhu et al. (Table 1) [78], remained nearly unchanged after six months of aging. However, the recovery rate in air decreased significantly.

#### 3.2.2. Pt-Decorated ZnO

Analogous to Pt-decorated ZnO, the ZnO morphology impacts the sensing properties of Pt-decorated ZnO-based H_2_ sensors.

**Figure 9:**
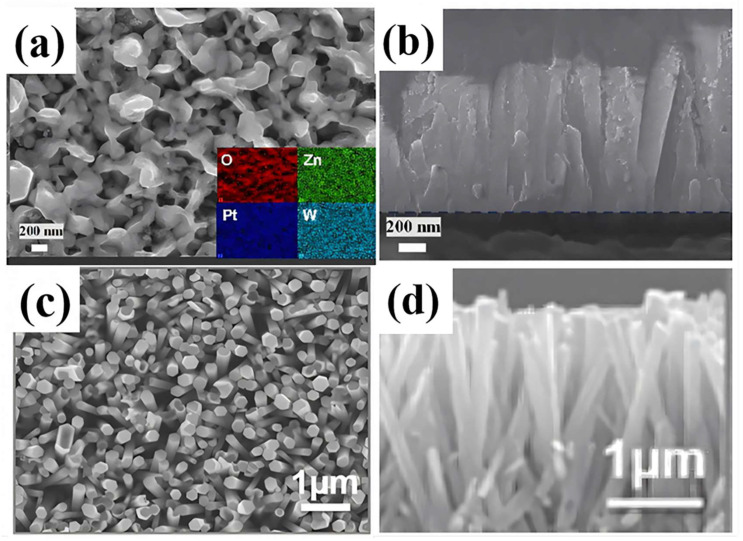
(**a**,**b**) SEM image and cross-sectional SEM of WPt_0.25_/ZnO and the elemental distribution. [80] Reprinted with permission from Elsevier, copyright 2024. (**c**) Field emission SEM images of the optimal ZNT/G. (**d**) The TEM microstructure cross sections of the optimal ZNT/G [81]. Reprinted with permission from [81]. Copyright {2017} American Chemical Society.

In the work conducted by Uddin et al. (Table 1) [79], the ZnO morphology was optimized through rapid thermal annealing, resulting in an optimal pencil-like topography sensing material suitable for industrial applications up to 300 °C. Additionally, Tan et al. [80] directionally grew ZnO NR arrays on glass substrates and subsequently coated them with WO_3_/Pt (Figure 9a,b). Benefiting from the advantages of effective carrier transport in nanoarrays, the high catalytic efficiency of Pt clusters, and the work function of WO_3_ NPs, the optimal materials exhibited exceptional H_2_ sensing performances, achieving a response of 61.5 to 100 ppm H_2_ with response and recovery times of 19 and 81 s, respectively.

Furthermore, ZnO NTs (ZNTs) are also employed in gas sensing applications due to their advantages of having a larger surface area, higher surface oxygen vacancy concentration, and an elevated surface-to-volume ratio [81]. However, ZNTs suffer from limitations such as poor selectivity, stability, and operating temperatures, which impede their progress in gas sensing. These challenges can be addressed by the preparation of hybrid nanocomposites [110]. Kathiravan et al. (Table 1) [81] documented the innovative nanostructural interfaces of self-assembled hierarchical ZnO NTs/graphene (ZNT/G) composites by systematically modulating the growth times of ZNTs on graphene substrates (Figure 9c,d). The optimal ZNT/G sensor demonstrated exceptional repeatability, reliability, and sustained long-term stability over a period of 90 days during hydrogenation/dehydrogenation cycles [81]. This superior performance was attributed to the formation of a robust metallized region at the ZNT/G interface, facilitated by the inner and outer surfaces of the ZNTs, which collectively established a multifaceted depletion layer. Vivekanandan et al. (Table 1) [82] constructed a hybrid structure comprising MoS_2_-incorporated ZnO hollow NTs (MoS_2_-HIZNTs). This hybrid nanostructure was synthesized through a simple soft-chemical method involving the etching of ZNTs in an aqueous solution with MoS_2_ serving as an inducible candidate. The resulting MoS_2_-HIZNT material exhibits a unique labyrinth-like structure, leading to exceptional H_2_ sensing performance at RT. The enhanced surface area of MoS_2_-HIZNTs facilitates the adsorption of more gas ions, resulting in a linear increase in oxygen vacancies and surface-active sites.

**Figure 10:**
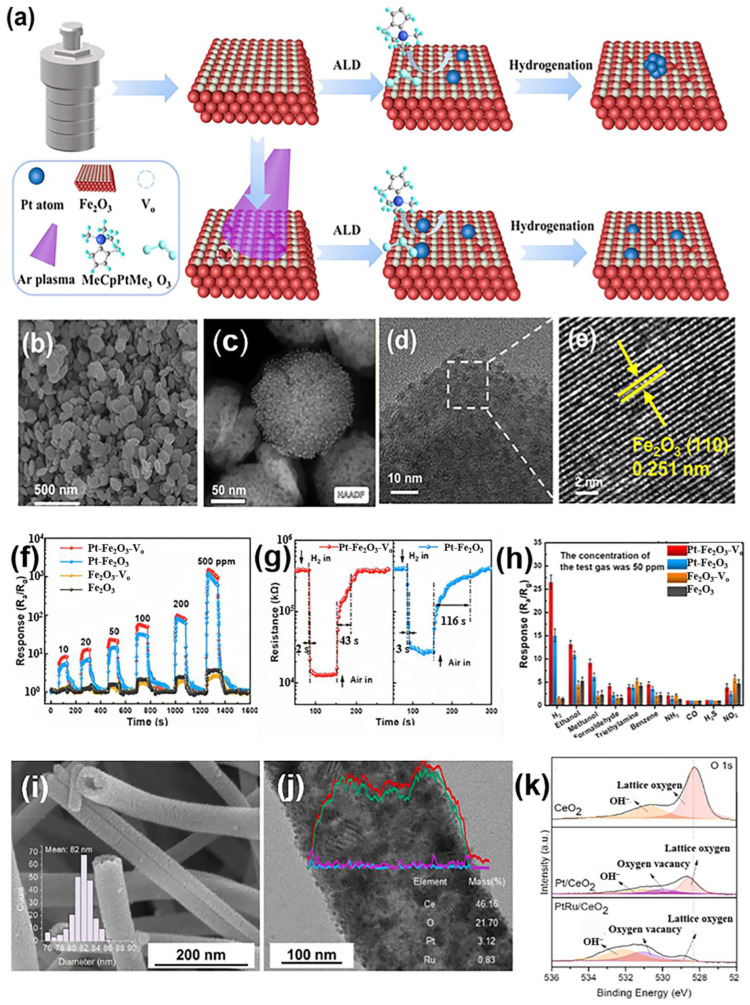
(**a**) Synthesis of Pt-loaded Fe_2_O_3_ NSs (Pt–Fe_2_O_3_–Vo). Characterization of Pt–Fe_2_O_3_–Vo [85]: (**b**) SEM images [83]; (**c**) EDX elemental mappings [83]; (**d**,**e**) HRTEM images [83]. (**f**) Dynamic curve of sensor response at different H_2_ concentrations [83]. (**g**) Response–recovery time of Pt–Fe_2_O_3_–Vo and Pt–Fe_2_O_3_ sensors to 50 ppm of H_2_ at 240 °C [83]. (**h**) Selectivity of the sensors to different gases [83]. Reprinted with permission from [83]. Copyright {2024} American Chemical Society. (**i**) FESEM image of Ce electrospun fiber after calcination at 500 °C with inset showing diameter distribution (mean: 82 nm) [84]. (**j**) HRTEM image of PtRu/CeO_2_ with energy dispersive spectroscopy mapping [84]. (**k**) X-ray photoelectron spectroscopy of O 1s [84]. Reprinted with permission from Elsevier, copyright 2024.

As discussed in Section 3.1.2, the oxygen defects in MOSs nanomaterials possess a unique electronic structure and unsaturated coordination environment, which facilitates molecular adsorption and electron transfer in sensing reactions [111]. In addition to ZnO, both iron(III) oxide (Fe_2_O₃) [112] and CeO_2_ can enhance their H_2_ sensing performances through the introduction of oxygen defects. Zhang et al. (Table 1) [83] reported a stable H_2_ sensor based on Pt single atoms (Pt SA) anchored to oxygen-rich vacancies on Fe_2_O₃ NSs (Pt–Fe_2_O₃–Vo) (Figure 10). The surface oxygen vacancies were introduced in the last step under the reducing condition (Figure 10a). Gas sensing studies revealed that at an optimal temperature of 240 °C, the sensor response of Pt–Fe_2_O₃–Vo was improved by a factor of 17 compared to pure Fe_2_O₃ (Figure 10f), with an ultra-fast response time of 2 s (Figure 10g). It also delivered excellent selectivity, as illustrated in Figure 10h. The exceptional sensing performance of Pt–Fe_2_O₃–Vo is attributed to the unique morphology (Figure 10b–e), which favored oxygen spillover. Experimental and density functional theory (DFT) calculations [113] demonstrated that the Pt-Fe atomic site at the oxygen vacancy exhibits higher binding energy, leading to a strong electronic interaction between Pt and the Fe_2_O₃ surface, which stabilizes the Pt SA and enhances the sensing performance. CeO_2_, characterized by numerous intrinsic defects, possesses various intriguing properties such as oxygen-rich defects, significant redox properties, high oxygen storage capacity, and the ability to absorb and release oxygen through the conversion between Ce_3_⁺ and Ce_4_⁺. These attributes of CeO_2_ are highly promising for exceptional gas sensing performance. Kim et al. (Table 1) [84] developed CeO_2_ hollow NFs (Figure 10i,j) through electrospinning to enhance the interaction between oxygen vacancies (Figure 10k) and H_2_ on PtRu, resulting in higher selectivity and a broader detection range (100 ppm to 50%) compared to the CeO_2_ and Pt/CeO_2_.

#### 3.2.3. Other Noble-Metal-Decorated MOSs

In addition to Pd and Pt, other noble-metal-decorated MOS nanomaterials also exhibit excellent H_2_ sensing performance, such as Au, Ag, Ir, etc.

Hyodo et al. [114] fabricated Au(n)/Pt/TiO_2_ for H_2_ sensing. They found that water molecules and/or hydroxyl groups adsorbed on the surface played a crucial role in increasing the H_2_ adsorption and dissociation on the surface, thereby enhancing H_2_ sensing performances.

**Figure 11:**
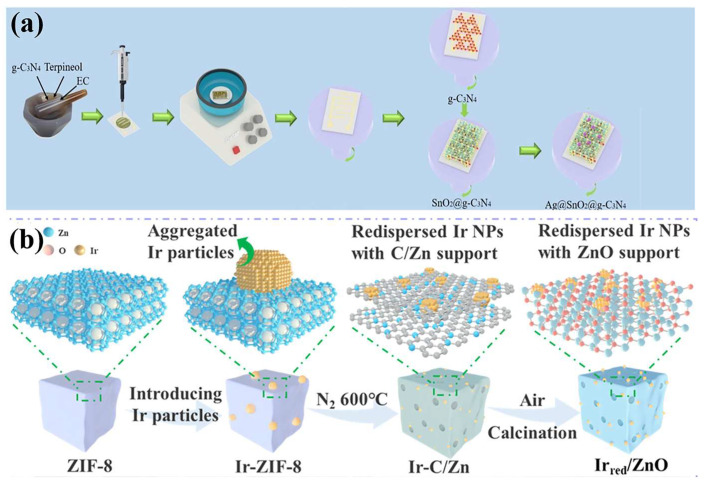
(**a**) Fabrication process of Ag@SnO_2_@g-C_3_N_4_ [85]. Reprinted with permission from Elsevier, copyright 2024. (**b**) Schematic illustration of the Ir_red_/ZnO synthetic route [86]. Reprinted with permission from [86]. Copyright {2024} American Chemical Society.

Shao et al. (Table 1) [85] introduced a novel H_2_ sensor featuring a sandwich structure that incorporates Ag. This structure comprises a catalytic sensitization layer composed of Ag NPs, a gas sensing layer of SnO_2_, and an electron supply layer of graphitic carbon nitride (g-C_3_N_4_), collectively referred to as the catalytic-sensitization-layer gas-sensing-layer electron-supply-layer (CSE) configuration (Figure 11a). The optimal Ag@SnO_2_@g-C_3_N_4_ material exhibited a detection limit of 30 ppb, with response and recovery times of 7 s and 9.7 s, respectively, as well as remarkable long-term stability. Guo et al. (Table 1) [86] achieved successful redispersion of Ir NPs through carbon-assisted pyrolysis, thereby enhancing the activity and stability of H_2_ sensors. They used a typical metal–organic framework [115,116,117], ZIF-8, as the precursor to obtain the carbon-decorated ZnO via the annealing in N_2_ and calcination in air (Figure 11b). The obtained materials inherited the porous structure from the ZIF-8 and showed a large specific area, which guaranteed rich reactive sites. By improving the dispersion and uniformity of Ir NPs, the catalytic performance of the material was significantly enhanced. The redispersed Ir NPs possess a larger active surface area, which is pivotal in enhancing the performance of the H_2_ sensor.

## 4. Conclusions

Chemoresistive H_2_ sensors play a pivotal role in various fields, including the hydrogen energy industry, environmental monitoring, and medical diagnosis. The development of cost-effective sensing materials is crucial for advancing these applications. MOSs have emerged as potential candidates for H_2_ sensing, yet their inherent sensing performance remains limited. To enhance their performances, the introduction of noble metals to create noble-metal-decorated MOSs sensing materials has become a widely adopted strategy.

Despite recent extensive research and reviews documenting progress in this field, a comprehensive analysis specifically addressing the rational design of sensing materials to optimize the overall performance of noble-metal-decorated MOS-based chemoresistive H_2_ sensors is lacking. This review summarizes the recent research advancements in noble-metal-decorated MOSs-based H_2_ sensing materials. We comprehensively consolidate and analyze the strategies utilized in the literature, particularly those reported within the last three years, to furnish a foundational comprehension of the rationale underlying the design of highly efficient chemoresistive H_2_ sensors. Current research efforts in this field primarily focus on noble metals such as Pd, Pt, Au, Ag, and Ir, while the MOSs are mainly SnO_2_, ZnO, and NiO. Enhancing the dispersion and uniformity of noble metal NPs is anticipated to boost the catalytic properties of these materials. Furthermore, manipulating the morphology of noble-metal-decorated MOSs-based sensing materials is another promising strategy. Morphologies that can yield a high specific surface area, such as NWs [5,58,59], NSs [60], NFs [59], NRs [64], and NTs [66], are preferred due to the abundance of reaction sites. Additionally, the construction of MOSs into heterojunctions, such as p-n-type NiO-Fe_2_O_3_ and PdO-WO_3_, or the incorporation of other components like G and rGO, can further improve the H_2_ sensing performance of the material. These strategies collectively offer significant potential for enhancing the sensing capabilities of noble-metal-decorated MOSs in H_2_ detection applications.

The incorporation of oxygen vacancies and the precise modulation of their concentrations within MOSs constitute a widely employed strategy. Reducible oxides, exemplified by ZnO, CeO_2_, and Fe_2_O_3_, are typically utilized for this purpose. The creation of oxygen defects is commonly achieved through gas reduction and calcination treatments. However, these methodologies often suffer from complex or dangerous conditions, including high temperatures and reducing atmospheres. Consequently, the development of a safe and easily operated method for controlling oxygen vacancy concentration at RT utilizing an appropriate reducing agent is important. Ascorbic acid has emerged as a promising candidate for facilitating the formation of both surface-bound and intrinsic hydroxyl groups in the context of oxygen vacancy modulation. The adoption of the hydroxyl-oxygen vacancy model for sensing materials heralds a novel approach to synthesizing high-performance gas sensors. Nonetheless, the attainment of remarkably high response values at RT is accompanied by an elongation in the recovery time to the initial baseline. The challenge of reducing recovery time while maintaining high sensor responsiveness remains an area requiring further exploration and investigation.

The H_2_ sensing performances of perovskite materials such as BaTiO_3_, which are also prone to oxygen vacancies, have not been widely explored like materials such as SnO_2_ and WO_3_, despite their physical and chemical properties making them interesting candidates for gas sensing applications [118]. Perhaps due to its complex H_2_ sensing mechanism, it may undergo a phase transition from ferroelectric to paraelectric within the temperature range of H_2_ sensing. Multiple influencing factors make it complex to regulate their H_2_ sensing performances. Fully understanding the H_2_ sensing mechanism of such systems can help improve the H_2_ sensing performance of such materials.

Multi-sensing mode represents one of the most promising avenues for future development in gas sensing technology. Gas sensors equipped with a single sensing mode are frequently constrained by their operational principles and the properties of sensitive materials, potentially leading to false positives or negatives in specific conditions. By integrating sensors based on diverse principles, such as electrochemical, optical, thermal, and chemical impedance [119], the multi-sensing mode facilitates multi-dimensional detection of target gases. This integration significantly enhances detection accuracy and reliability, mitigating the risks associated with false positives and negatives. For instance, combining the gasochromic properties of WO_3_ with the chemoresistive characteristics of SnO_2_ enables the design of an H_2_ sensor with a dual detection mode, thereby improving its detection accuracy and reliability. Furthermore, under the multi-sensing mode, algorithms such as machine learning [120] can be employed to process and analyze this data by amalgamating multiple sensors and gathering extensive data [121,122,123,124,125], enabling precise predictions of gas concentration and real-time detection under complex environments.

Although chemoresistive H_2_ sensors based on noble-metal-decorated MOSs have demonstrated their high responsiveness and low detection limits, they also face many challenges, such as performance issues in high-temperature environments, poor selectivity, and humidity-dependent response. Breakthroughs on these issues are needed in the future.

In summary, the future development of noble-metal-decorated MOSs-based H_2_ sensors may follow the directions of cost-effectiveness, intelligence, and integration.

## Data Availability

The original contributions presented in this study are included in the article material. Further inquiries can be directed to the corresponding authors.
